# Identification and characterization of novel enhanced cell penetrating peptides for anti-cancer cargo delivery

**DOI:** 10.18632/oncotarget.23179

**Published:** 2017-12-11

**Authors:** Xiguang Zhang, Jean Yves Brossas, Christophe Parizot, Jean Marc Zini, Angelita Rebollo

**Affiliations:** ^1^ CIMI Paris, Inserm 1135, UPMC, 75013 Paris, France; ^2^ Hôpital Pitié Salpêtrière, AP-HP, Department of Immunology, 75013 Paris, France; ^3^ Hôpital Saint Louis, AP HP, 75010 Paris, France

**Keywords:** cell penetrating peptides, peptide internalization, peptide stablity

## Abstract

Cell penetrating peptides (CPP) are able cross the membrane and to transport cargos, presenting a great potential in drug delivery and diagnosis. In this paper, we have identified novel natural or synthetic CPPs. We have validated their rapid and efficient time and dose-dependent penetration, the absence of toxicity, the intracellular localization and the stability to proteases degradation, one of the main bottlenecks of peptides. Moreover, we have associate a cargo (an interfering peptide blocking the association of the serine/threonine phosphatase PP2A to its inhibitor, the oncogene SET) to the new generated shuttles and showed that they new bi-functional peptides keep the original properties of the shuttle and, in addition, are able to induce apoptosis due to the properties of the cargo. The CPPs identified in this study have promising perspectives for future anti-cancer drug delivery.

## INTRODUCTION

One of the obstacles to the use of therapeutic molecules having intracellular targets is they low penetration through the membrane. The best way to solve this problem is the use of cell penetrating peptides (CPP), molecules capable to cross the cell membrane. They can transport a great variety of cargos into the cell in terms of size and nature [1–3]. Several studies tried to design novel CPP with greater efficacy and selectivity. Since Tat (CPP with high number of basic residues), Wender and co workers showed that poly Arginine peptide is an efficient CPP [4]. Several groups have synthesized oligo arginines of different sizes and studied their internalization and efficiency [5].

Electrostatic interactions between the positive charges in the cell membrane surface are important for the internalization. The presence of basic residues in the CPP showed that arginine residues are important for internalization of the CPP [6–8]. From these studies, the CPP RW9 (RRWWRRWRR) was designed and shown to have good cellular uptake. When tryptophan residues were replaced by leucine residues, the peptide was not internalized [9, 10]. In addition, it has been shown that the R9 oligo arginine CPP is able to internalized at similar level as RW9.

So far, a number of peptide sequences have been reported as CPPs. They can be classified according to physicochemical characteristics, such as basic/anphiphilic and hydrophobic. CPPs commonly have high content of positively charged residues such as arginine and lysine, that may influence cellular internalization [9, 11–14].

Although many CPP have been identified so far, most of then have shown low internalization efficiency and low stability in contact to plasma proteases. We have described the use of Mut3DPT [15], that show favourable parameters of resistance to protease degradation. In this study, we have used experimental approach to identify novel, efficient and stable CPP. Their cell penetration properties and protease-resistance were validated experimentally on cell lines and primary cells using flow cytometry (FACS), microscopy and mass spectrometry. Further, they were associated to a cargo to estimate its impact on the original properties of the CPP.

## RESULTS

### Rational design of new cell penetrating peptides (CPP)

We have recently published the use of a CPP named Mut3DPT [15, 16]. This CPP shows favourable parameters of resistance to protease degradation. We decided to generate new CPPs with enhanced penetration and stability, compared to the original Mut3DPT shuttle and keeping equilibrium among penetration, solubility and toxicity. As shown in Table 1, the shuttle Mut4DPT derivates from the sequence of Mut3DPT in which we have mutated 3 residues, lysine (K), alanine (A) and glutamic acid (E) by tryptophan (W), that have been describes as amino acids that play an important role in delivery efficiency.

**Table 1:** Characteristics of the shuttles used in this study

Peptide ID	Peptides Sequence	Arginine	Tryptophan	Lysine	Charge	PI	Mol Wt
Mut3DPT	VKKKKIKAEIKI	0	0	6	5.00	10.3	1426.05
Mut4DPT	VKKWKIKWWIKI	0	3	5	5.00	10.6	1656.33
Mut5DPT	KKWKKWKKWKK	0	3	8	8.00	10.85	1602.21
Mut6DPT	RRWRRWRRWRR	8	3	0	8.00	12.85	1826.29
Mut7DPT	KKKKKWKKWKKK	0	2	10	10.00	10.96	1672.36

Mut5DPT is a synthetic sequence of lysine residues in which we play with the density of tryptophan residues and their position in the sequence. Mut6DPT was designed to be compared to Mut5DPT to analyze the role in penetration of arginine (R) compared to lysine (K). The electrostatic interaction is made in one case by lysine and in the other by arginine (R). Mut7DPT is a derivate of Mut5 in which we change the position of tryptophan and lysine residues. This peptide has two extra lysine residues (Table 1).

### Analyse of the cytotoxicity of the peptides

**Figure 1:**
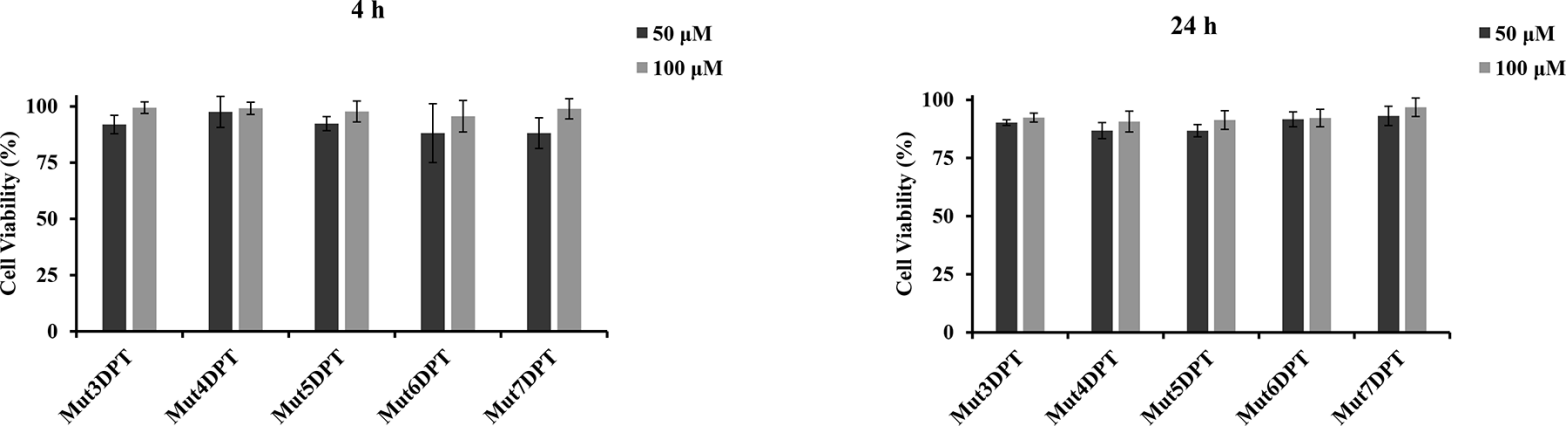
Analysis of citotoxicity of the new shuttles Cells (MCF-7) were cultured in 96 well plates with two different concentrations of shuttle for 4 or 24 h at 37°C. Cell viability was measured by WST assay. Viability of the control non treated cells was used as 100%. Viability of treated cells was represented as percentage of the control. Standard deviation of triplicates of two independent experiments is shown.

Before testing the stability and the penetration of the shuttles, we analyzed their toxicity in MDA-MB231 cell line at two different concentrations and time. All shuttles were added to the cells at concentration of 50 and 100 µM and the toxicity was analyzed upon 4 or 24 h of incubation. As shown in Figure 1, none of the shuttles show toxicity, independent of the concentration or the time of incubation, even at the higher concentration of 100 µM .

### Quantification of internalized predicted cell penetrating peptides

**Figure 2:**
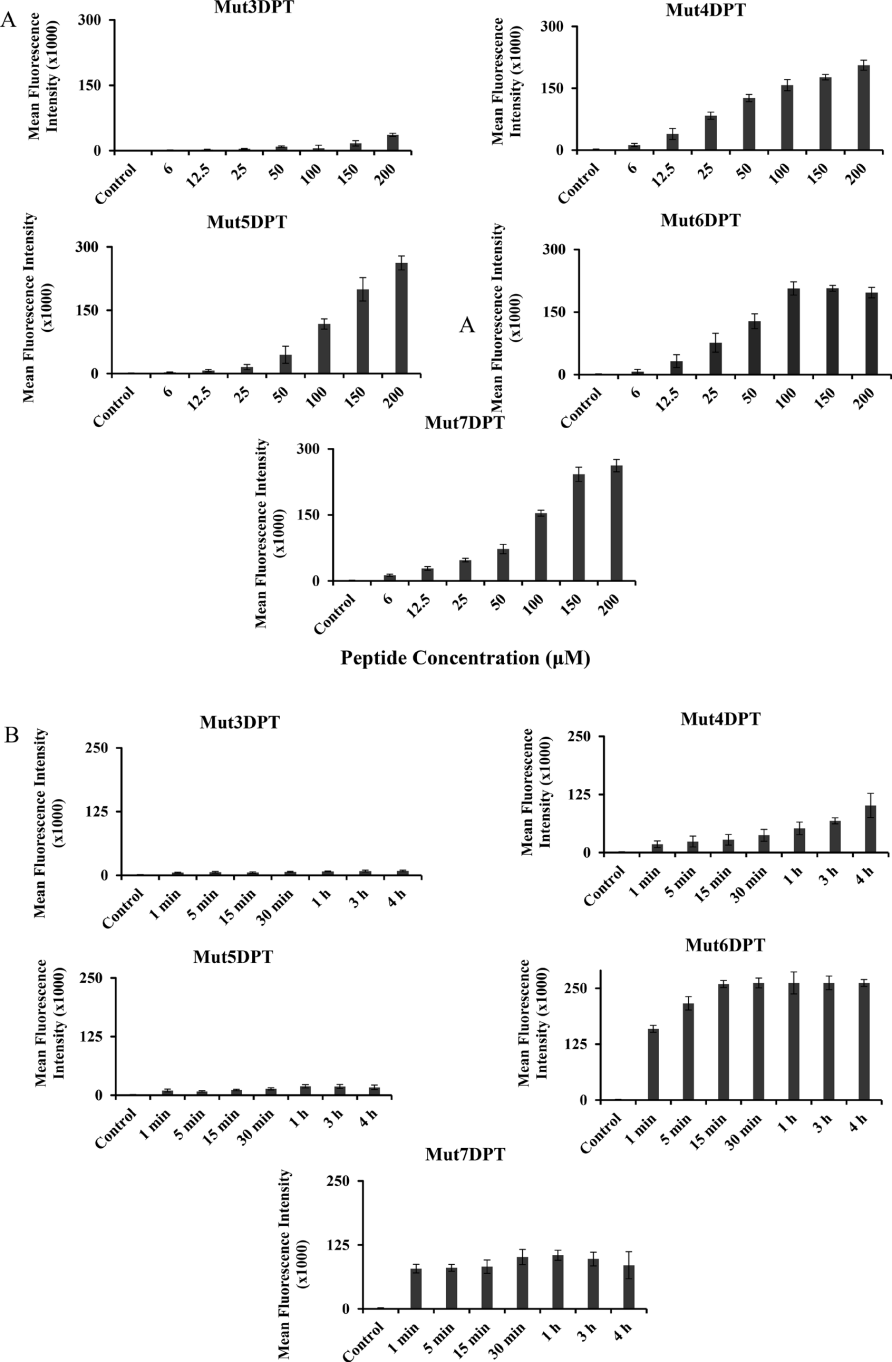
Concentration and time-dependent internalization of FITC-labelled shuttles (**A**) MCF-7 cells were incubated 4 h with different concentrations of FITC-labelled shuttle. The mean fluorescence intensity was detected by flow cytometry and compared to control non treated cells. (**B**) MCF-7 cells were incubated 4 h with 50 µM of the FITC-labelled shuttles for different periods of time. The mean fluorescence intensity was detected by flow cytometry. Non treated cells were used as control. Standard deviation is shown.

We evaluated whether the predicted CPPs were able to internalize into cells. The peptides were labelled with FITC and their internalization was analyzed by FACS. Mut3DPT, our previously described CPP, was used as a control to compare the internalization. MCF7 cells were treated with FITC-labelled peptides at different concentrations for 4h and then, internalization analyzed by FACS. Figure 2A shows the internalization of the shuttles, compared to Mut3DPT peptide control. Mut4DPT, Mut5DPT, Mut6DPT and Mut7DPT show similar concentration dependent fluorescence intensity, compared to the control peptide, Mut3DPT, that shows much lower fluorescence intensity into the cells. Similar results were obtained using T cell line Jurkat (data not shown). We next analyzed the effect of time incubation in the internalization of a fixed shuttles concentration. Among them, Mut6DPT shows to be the CPP with an extremely rapid internalization (Figure 2B) reaching a plateau upon 15 min of incubation. Mut4DPT and Mut5DPT show similar time kinetic of internalization and stronger than in the control Mut3DPT shuttle. Mut7DPT shows higher time-dependent internalization that Mut4DPT and Mut5DPT. Taken together, all the new generated sequences are CPP and show more favourable internalization than the control Mut3DPT shuttle.

### Structural analysis of new shuttles

In order to understand why all the analyzed peptides show different internalization efficiency, even if they have similar charge (Mut3DPT/Mut4DPT and Mut5DPT/Mut6DPT) and the same number of arginine residues (Mut3DPT, Mut4DPT, Mut5DPT and Mut7DPT) (Table 1), we predicted the structure using PEP-FOLD.3. Tryptophans have been shown to have a strong preference for interactions at the membrane and several studies have proved its role in internalization. So, the presence of tryptophan in our shuttles appears to be the predominant factor contributing to internalization properties compared to the control shuttle Mut3DPT.

**Figure 3:**
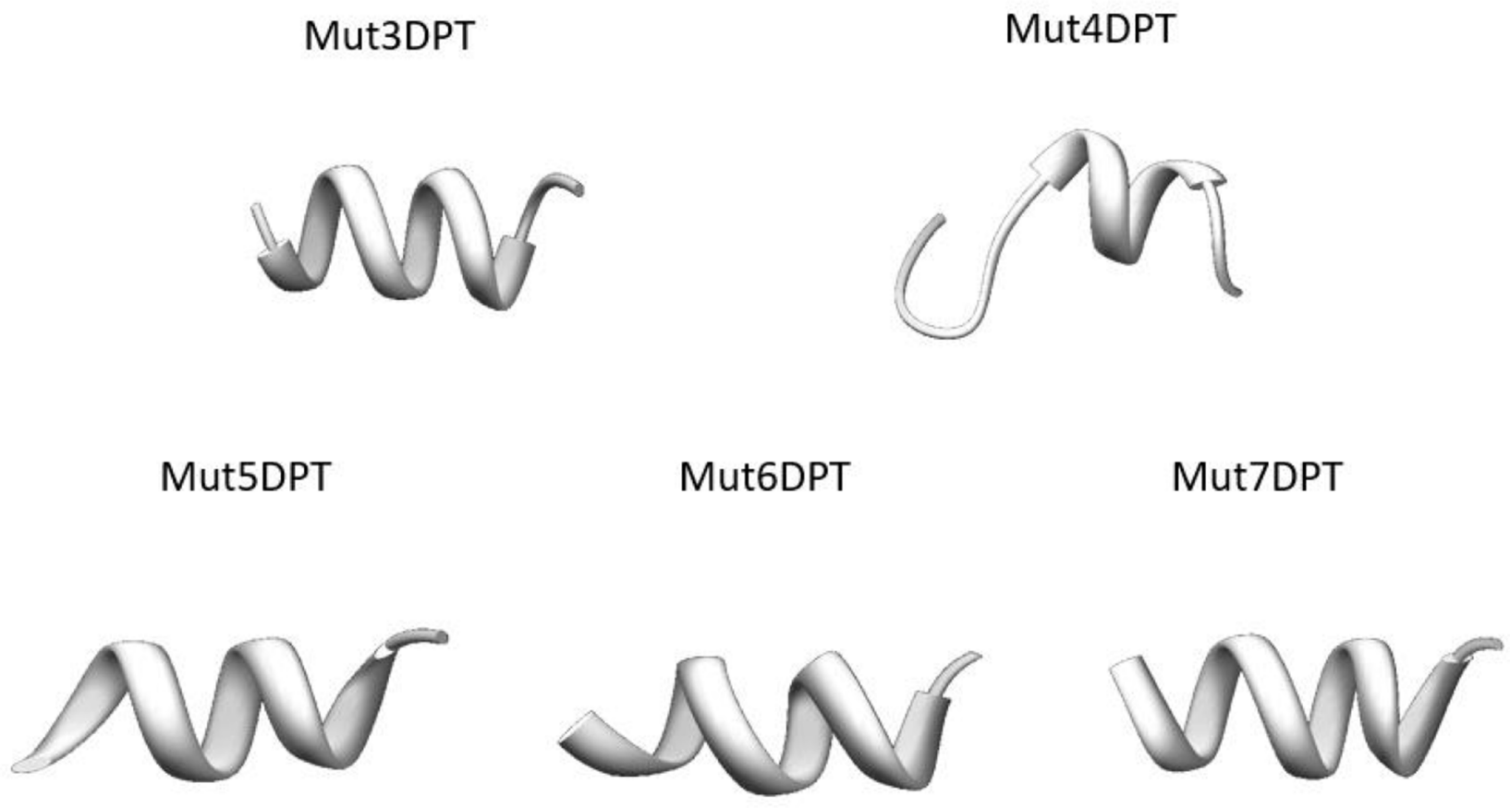
Tertiary structure of the new generated shuttles was predicted using PEP-FOLD

Several reports have suggested that structure of the peptides may play a role in membrane internalization, suggesting that peptides with helical structure internalize more efficiently than shuttles with random structure. The 3D structure of all the new shuttles (Mut3DPT to Mut7DPT) shows a well defined helix conformation (Figure 3), although less clear for Mut4DPT. This result may suggest that the higher internalization could be due to the presence of tryptophan residues in the sequence of the shuttle and the helix structure.

Interestingly, Mut5DPT and Mut6DPT are similar in terms of sequence, structure and number of tryptophan residues in the sequence, however, their internalization efficiency is very different (Figure 2A–2B). It has been published that, in addition to the number of tryptophan, the spacing and the location in the shuttle may also affect the internalization. In the case of Mut5DPT and Mut6DPT, the spacing and the number of tryptophan is the same, suggesting that arginine residues are more efficient that lysine for electrostatic interactions with the membrane.

### Intracellular localization of the shuttles

**Figure 4:**
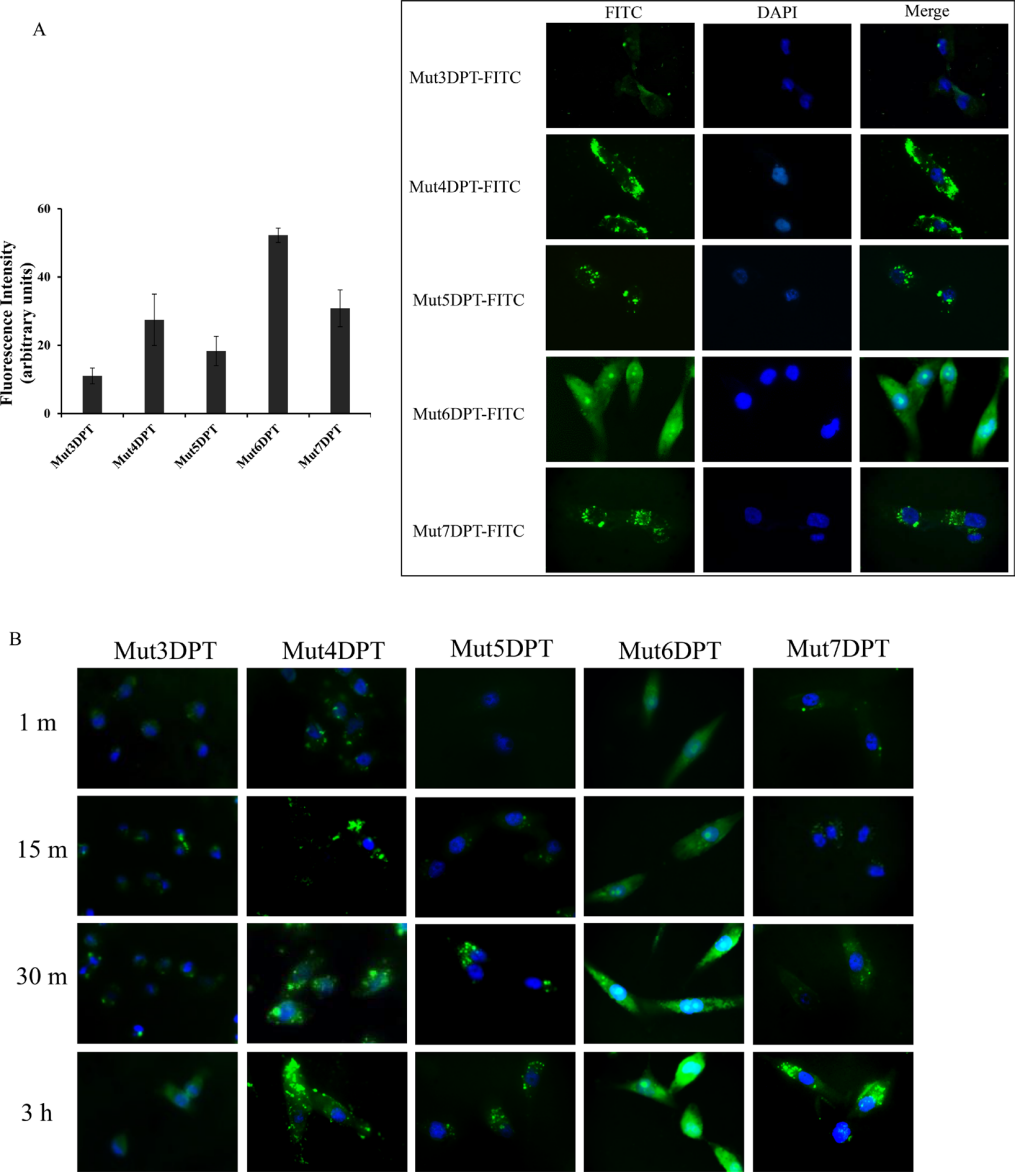
Intracellular localization of FITC-labelled shuttles (**A**) MCF-7 cells were grown on coverslips and incubated 1 h at 37°C with 25 µM of FITC-labelled peptides. Cells were washed 3 times with PBS, fixed with 4% paraphormaldeyde (PFA) and analyzed by fluorescence microscopy. Quantification of the microscopy images is shown. (**B**) MCF7 were grown as above and incubated for different periods of time at 37°C with 25 µM of FITC-labelled shuttles. Cells were treated as above and analyzed by fluorescence microscopy.

To confirm the FACS results and to visualize the intracellular distribution of shuttles, we stained MCF7 cells with FITC-labelled peptides and analyzed the penetration by fluorescence microscopy following 1 h of incubation at a concentration of 25 µM (Figure 4). The staining patter varies among the different peptides. We observed a punctuate staining for peptides Mut5DPT and Mut7DPT, located in the cytosol and excluded from the nucleus. Mut4DPT and Mut6 DPT show a diffuse and homogeneous staining through the cytoplasma and nucleus for Mut6DPT and preferentially cytoplasma staining for Mut4DPT. The diffuse intracellular staining might indicate efficient escape from endocytotic vesicles. The control Mut3DPT shows a weak staining, confirming the results obtained by FACS. Figure 4B shows a time kinetic of shuttles penetration. Mut6DPT shows a rapid penetration with detection of intracellular fluorescence upon 1 min of incubation with the peptide.

### Detection of the shuttles into the cell by mass spectrometry (MS)

**Figure 5:**
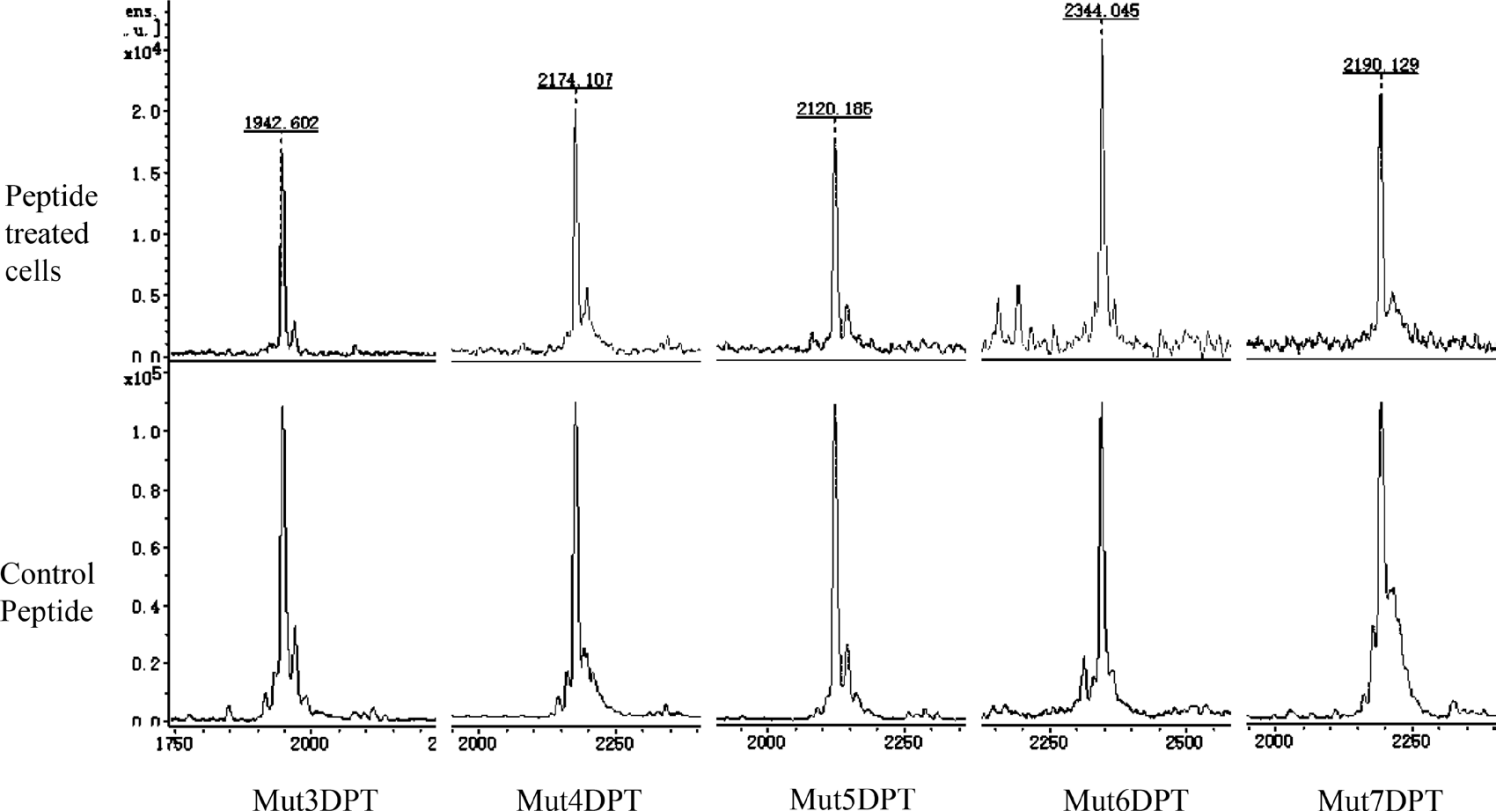
Intracellular detection by mass spectrometry (MS) of the shuttles MCF-7 cells were cultured 4 h with 100 µM of each shuttle. After several washing steps, they were lysed and the extracts centrifuged. The intracellular peptide was detected by MS. Pure peptide was used as a control of molecular weght.

In order to confirm the internalization of the shuttles, detected by FACS and microscopy, we used a complementary approach, MS, for intracellular detection of the shuttles. Cells were treated 4 h with 100 µM of each peptide and then lysed as described in Materials and Methods section of the manuscript. The cytoplasmic extracts were analyzed by MS in order to detect the presence of the peptides into the cells. As shown in Figure 5, all the shuttles were detected in a non-degraded form in lysates of treated cells. We used pure peptide as internal control of the molecular weight of the peptides.

### Internalization of shuttles on primary cells

In addition to cell lines, we also tested the internalization of the new generated shuttles in B cells from healthy and tumoral primary cells, isolated from peripheral blood mononuclear cells (PBMC) from healthy donors (HD) or from chronic lymphocytic leukemia (CLL) patients.

**Figure 6:**
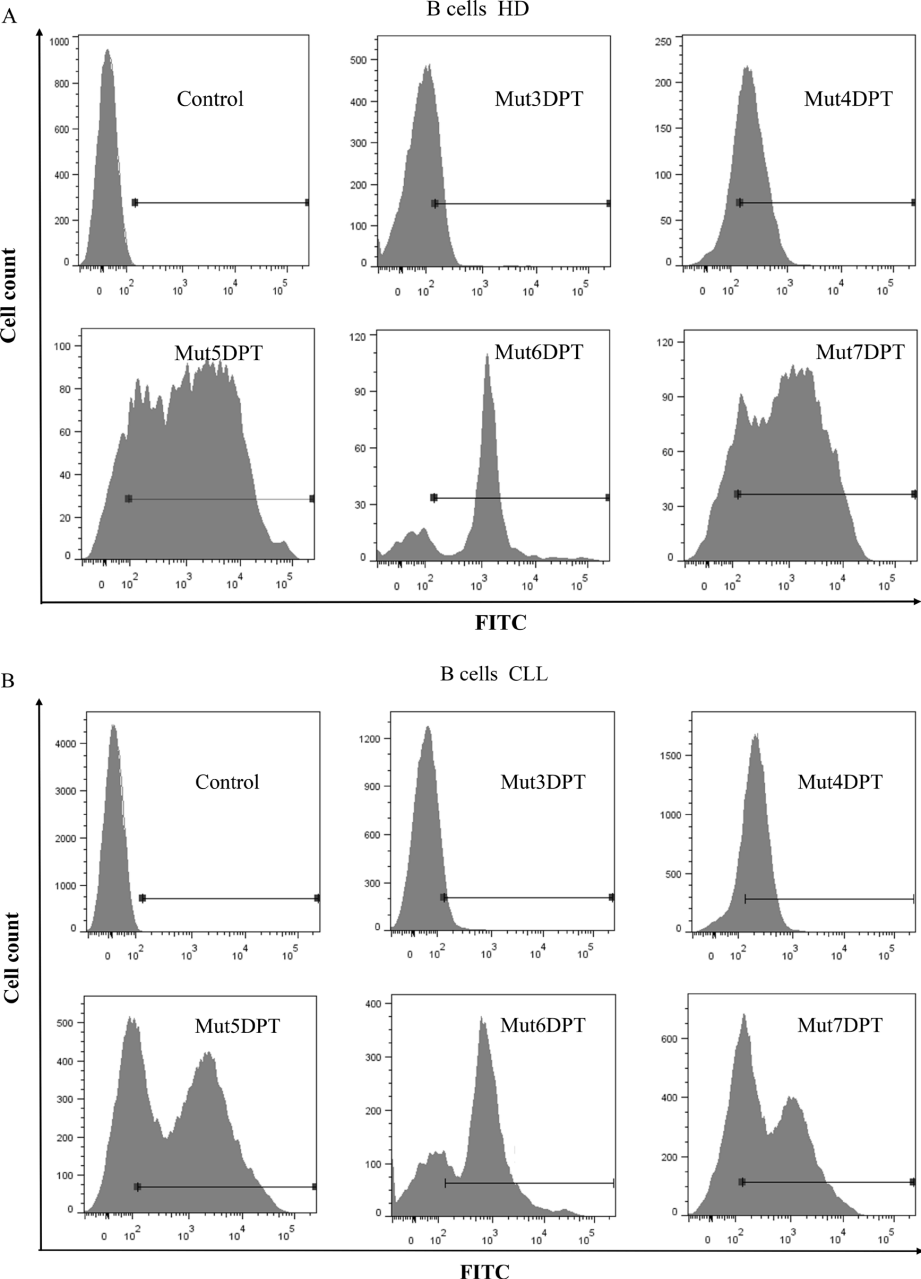
Internalization of FITC-labelled shuttles on healthy and tumoral primary B cells (**A**) B cells were isolated (anti-hCD19-APC antibody) from peripheral blood mononuclear cells (PBMC) of healthy donors (HD) or (**B**) chronic lymphocytic leukemia (CLL) patients. Cells were incubated 4 h with 100 µM of the FITC-labelled shuttles. The mean fluorescence intensity was detected by flow cytometry. Non treated cells were used as control.

PBMC from both were incubated with a concentration of 50 µM of all the shuttles for 4 h at 37°C. B cells were selected by staining with anti human CD19 antibody before FACS analysis. As illustrated on Figure 6A, the parental Mut3DPT-FITC peptide shows the lowest intensity of penetration detected by flow cytometry in B cells isolated from PBMC of healthy donors. The shuttle Mut4DPT shows a moderate internalization in B cells from healthy donor and Mut5DPT, Mut6DPT and Mut7DPT show the highest value of mean florescence intensity in healthy donors. Similar results were obtained when B cells were isolated from PBMC of CLL patients (Figure 6B). The control Mut3DPT shuttle shows the lowest mean fluorescence intensity, being Mut4DPT, Mut5DPT, Mut6DPT and Mut7DPT the shuttles with the highest level of internalization in B cells from CLL patients.

### Resistance of the new generated shuttles to proteases degradation

**Figure 7:**
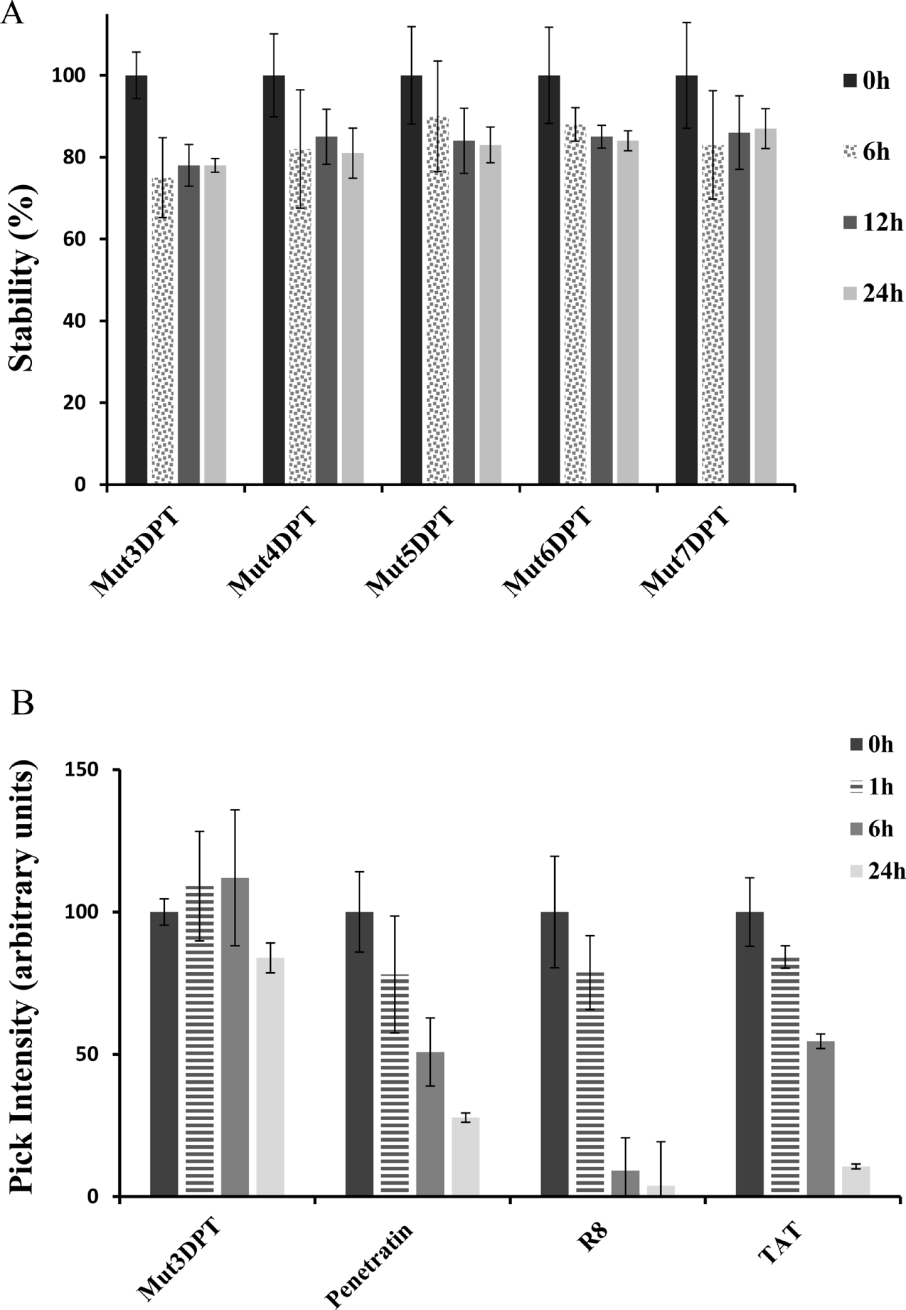
Stability of the new generated shuttles in human serum (**A**) The shuttles were incubated at 37°C in human serum for different periods of time and their integrity (percentage of intact peptide) was analyzed by mass spectrometry (MS). Every measurement was performed in triplicate. Standard deviation is shown. (**B**) The shuttles Tat, Penetatin and R8 were incubated as above and the integrity was analyzed by MS. Every measurement was performed in triplicate.

We further analyzed the stability to proteases degradation of the shuttles in human serum upon incubation at 37°C for different periods of time. All the shuttles were incubated with human serum and the resistance to proteases degradation analyzed by HPLC-MS. We did not detect significant degradation of the shuttles (Figure 7A) even upon 24 h of incubation un human serum. This result suggests that the new generated shuttles are as stable as the parental Mut3DPT shuttle, while having better profiles of internalization. The results of stability of the new generated shuttles were compared to other shuttles described in the literature such as Tat, Penetratin and R8. Figure 7B shows that penetratin, Tat and R8 are degraded by serum proteases and almost not detected by HPLC-MS upon 24 h of incubation, compared to our control shuttle Mut3DPT.

### Effect of association of a cargo on the structure of shuttles

We were interested in analyzing the contribution of a cargo to the modification of the shuttle structure and properties. We took advantage of the fact that we have mapped the binding site of the serine/threonine phosphatase PP2A to its physiological inhibitor, the SET oncoprotein. We have associated this interfering peptide to the shuttle Mut3DPT to generate a bi-functional peptide that can enter into the cell and block the PP2A/SET interaction (patent number WO2016156536). This bi-functional peptide is able to induce apoptosis *in vitro* in several cell lines, as well as in tumoral primary cells (Tian *et al.*, in press, International Journal of Peptide Research and Therapeutic). This complex is an interesting target for anti-cancer development strategies given his implication in tumoral transformation as well as hematological cancers.

**Figure 8:**
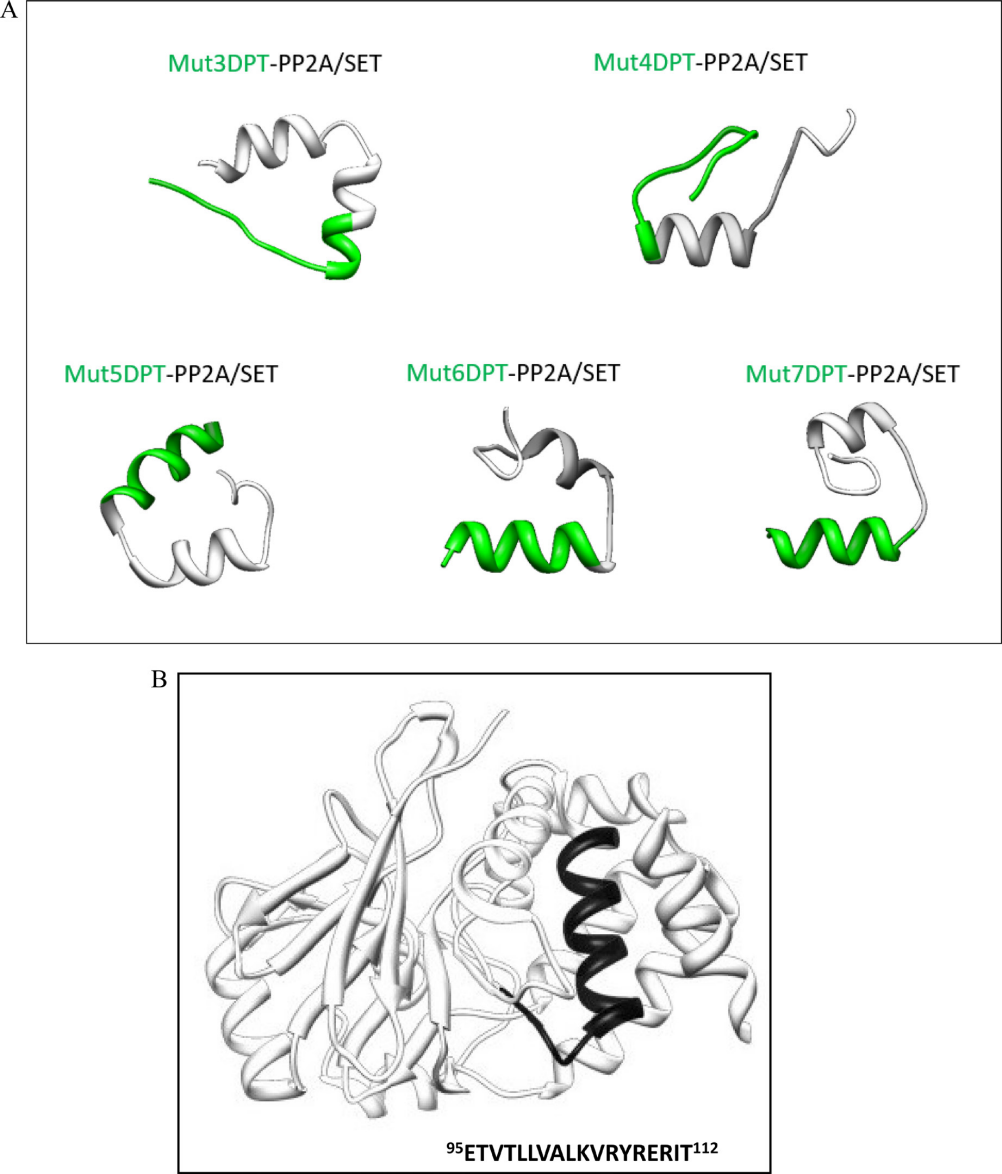
Tertiary structure of the shuttles associated to a cargo (**A**) The structure of the bi-functional peptides was predicted using PEP-FOLD. (**B**) Structure of the catalytic subunit of PP2A showing the binding site to SET

We have generated new bi-functional peptides composed of the new shuttles associated to the interfering peptide blocking PP2A/SET interaction (Table 2). The predicted 3D structure using the PEP-FOLD, which is a well-known in silico tool used to predict structure of small peptides, is shown on Figure 8A. Shuttle Mut5DPT-PP2A/SET and Mut6DPT-PP2A/SET have a defined helix conformation whereas Mut7DPT-PP2A/SET, Mut3DPT-PP2A/SET and Mut4DPT-PP2A/SET show a less well defined helix structure. When comparing the structure of the shuttles alone with the shuttles associated to a cargo, we observe that Mut3DPT and Mut7DPT loose the helix conformation. Figure 8B shows the structure of PP2A catalytic subunit with the binding site to SET is exposed.

**Table 2:** Characteristics of the shuttles associated to a cargo

Peptide ID	Peptides Sequence	Arginine	Tryptophan	Lysine	Charge	PI	Mol Wt
Mut3DPT-PP2A/SET	VKKKKIKAEIKIETVTLLVALKVRYRERIT	3	0	7	7.00	10.46	3568.91
Mut4DPT-PP2A/SET	VKKWKIKWWIKIETVTLLVALKVRYRERIT	3	3	6	7.00	10.71	3799.19
Mut5DPT-PP2A/SET	KKWKKWKKWKKETVTLLVALKVRYRERIT	3	3	9	10.00	10.87	3745.07
Mut6DPT-PP2A/SET	RRWRRWRRWRRETVTLLVALKVRYRERIT	11	3	1	10.00	12.25	3969.15
Mut7DPT-PP2A/SET	KKKKKWKKWKKKETVTLLVALKVRYRERIT	3	2	11	12.00	10.94	3815.22

### Impact of the cargo on the properties of shuttle

**Figure 9:**
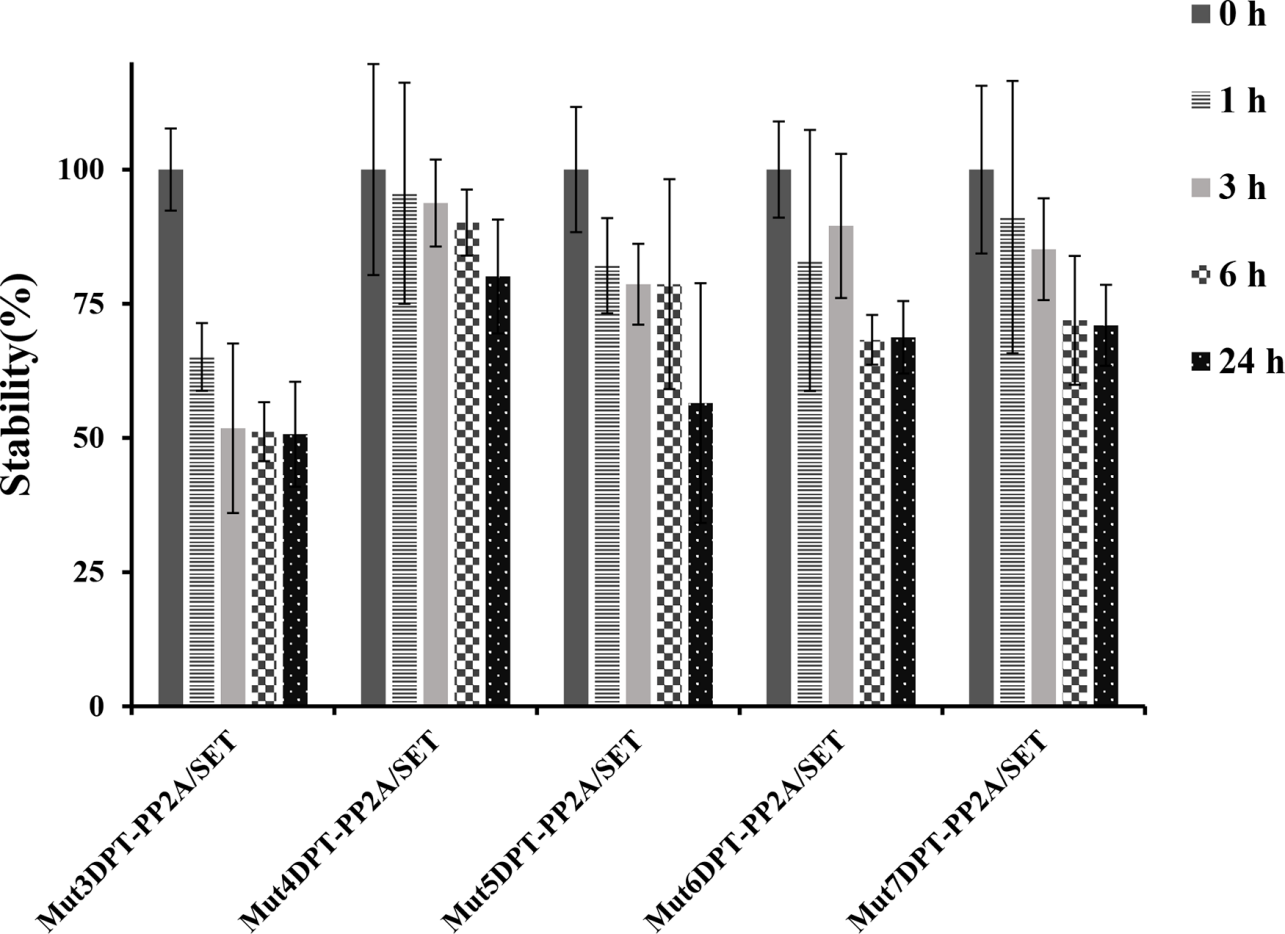
Stability in human serum of the shuttles associated to a cargo (PP2A/SET binding site) The shuttles associated to the cargo PP2A/SET were incubated at 37°C in human serum for different periods of time and their integrity (percentage of intact peptide) was analyzed by mass spectrometry (MS). Measurements were performed in triplicate and standard deviation is shown.

We first analyzed the impact on stability of the association of the interfering peptides PP2A/SET to the new generated shuttles. For this, the new generated bi-functional peptides (shuttles associated to the cargo PP2A/SET) were incubated with human serum for different periods of time. Figure 9 show that the association of a cargo to the shuttle Mut3DPT decrease the stability of the shuttle. The association of a cargo to the shuttles Mut4DPT, Mut6DPT and Mut7DPT does not significantly modify the stability, compared to the shuttle without cargo (Figure 7). A slightly degradation upon 24 h of incubation with human serum was observed for the shuttle Mut5DPT-PP2A/SET compared to the shuttle without cargo.

**Figure 10:**
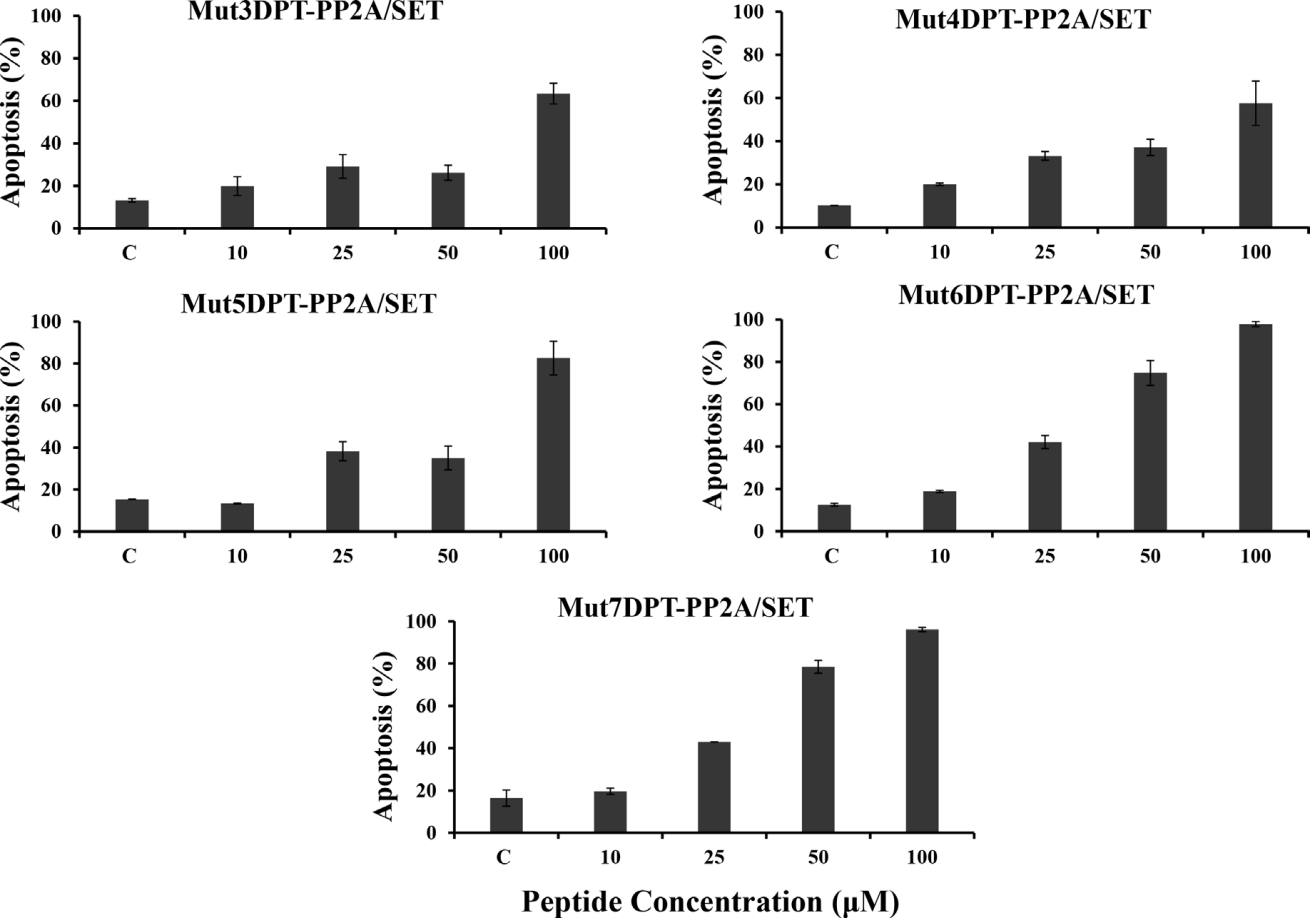
Detection of apoptosis induced by the shuttle associated to the cargo MCF-7 cells were cultured in the presence of different concentrations of peptides associated to the cargo (PP2A/SET binding site) for 24 h. Apoptosis was estimated by annexin-V-FITC staining. Non treated cells were used as control. Standard deviation is shown.

We further analyzed whether the apoptotic properties of the cargo were maintained when associated to the new generated shuttles. We have previously shown that the parental Mut3DPT-PP2A/SET bi-functional peptide induces apoptosis and tumor growth inhibition in CLL and lymphoma xenograft models (Tian et al, in press). We analyzed whether the apoptotic effect of the interfering peptide was affected when associated to a different shuttle (Figure 10). The cargo associated to the shuttle Mut4DPT show similar apoptotic effect that when associated to the control Mut3DPT shuttle. When the cargo was associated to the shuttles Mut5DPT, Mut6DPT and Mut7DPT, the level of apoptosis detected in MCF7 cells was higher, compared to the control shuttle Mut3DPT (Figure 10). Taken together, these results suggest that we have generated new shuttles with high capacity of penetration, stable to protease degradation and able to transport a cargo into the cell. The enhanced capacities of the shuttles are translated in enhanced properties of the cargo, as shown in the apoptotic effect of the cargo. Finally, the new generated shuttles can be used for anti-tumoral cargo delivery.

## DISCUSSION

The aim of this manuscript was to identify novel semi-synthetic or synthesis efficient cell penetrating peptides (CPP) and associate then to an anti-tumoral therapeutic cargo. We have combined residues of arginine or lysine with thryptophan to increase the penetration and, at the same time, keeping the properties of the control peptide Mut3DPT, the resistance to proteases degradation. Compared to other described shuttles such as Tat, penetratin or R8, our shuttles show higher stability. The size of the new generated CPPs range between 11 and 12 amino acids, a size that escape, normally, the presentation by MHC-II, which prefers longer peptides [17].

The flow cytometry, microscopy and mass spectrometry experiments revealed that all the generated shuttles (Mut4DPT to Mut7DPT) have enhanced cell penetration properties. None of the peptides showed toxicity, even at elevated doses such as 100 µM, suggesting that these peptides can be used for cargo delivery applications. All the shuttles showed enhanced penetration in adherent or suspension cells, as well as in primary cells, compared to the control shuttle Mut3DPT.

It has been shown that due to the presence of guanidinum groups, which interact with negative charges of the membrane, arginine residues play a more critical role in internalization that lysine residues [4, 18]. This is in agreement with our results in which we show that shuttle Mut6DPT, that contains 8 arginine residues is the best internalized shuttle. We can not exclude that other amino acids, such as tryptophan, may also contribute to the internalization efficiency. The orientation of arginine and tryptophan amino acids around the helix can also be responsible of a better penetration of a shuttle, which is in agreement with recent publications [19]. In addition, it has been shown that helical peptides are internalized more efficiently than peptides with random coil structures [20].

None of the shuttles show toxicity, so high penetration efficiency without toxicity makes these shuttles ideal candidates for cargo delivery and therapeutic application. Our designed shuttles show certain similarity in sequence to some types of anti-microbial peptides, which also have high content of arginine and tryptophan residues. It has been suggested that these two amino acids could contribute to anti-microbial properties [21]. However, our result suggest that our new shuttles are benign in mammalian cells, as described for the antimicrobial peptide LL-37, which has activity on bacteria without effect on mammalian cells [22].

Fluorescence microscopy experiments confirmed the differences in the extend of internalization of the new shuttles, which was also confirmed by flow cytometry and mass spectrometry. The potential of our shuttles to deliver a cargo was also analyzed using as a cargo the interfering peptide blocking the interaction between the serine/threonine phosphatase PP2A and its physiological inhibitor, the oncoprotein SET. PP2A is widely described as a tumor suppressor [23] and is a critical negative regulator of several oncoproteins. Inactivating mutations or decreased expression of PP2A subunits have been detected in a large variety of human malignancies [24]. Therefore, the tumor suppressor function of PP2A makes it an interesting target for novel anti-cancer therapies [25, 26]. The SET oncoprotein is the physiological inhibitor of PP2A. Elevated expression of SET has been linked to cell transformation, particularly in haematological malignancies [27]. SET inhibits PP2A by forming a complex with, so, many efforts trying to restore PP2A acrivity have focused on interfering PP2A/SET interactions [28, 29]. Using the PEPscan approach, we have mapped the binding site of PP2A to SET and generated a bi-functional peptide composed by the shuttle Mut3DPT associated to the sequence of interaction between PP2A and SET (patent WO2016057346, Tian *et al.*, in press).

Mut5DPT, Mut6DPT and Mut7DPT associated to the interfering peptide blocking PP2A/SET interaction, show high level of apoptosis when compared to the control shuttle associated to the same cargo, Mut3DPT PP2A/SET, suggesting that the original properties of the shuttles, the high penetration capability, is not modified when adding a cargo. In addition, the high penetration is translated into a high apoptotic effect mediated by the associated interfering peptide. Other characteristic of the shuttles that is maintained upon the association of a cargo is the resistance to proteases degradation, showing that the association of the interfering peptides does not have significant effect on the resistance of the shuttles to serum proteases degradation.

Taken together, the new shuttles are promising candidates for future applications as vectors for intracellular cargo delivery, already validated using as cargo the interfering peptide blocking the binding PP2A/SET.

## MATERIALS AND METHODS

### Peptide synthesis

Peptides were synthesized in an automated multiple peptide synthesizer with solid phase procedure and standard Fmoc chemistry. The purity and composition of the peptides were confirmed by reverse phase HPLC and by mass spectrometry (MS). The peptides were also synthesized whit a fluorochrome (FITC) in C-terminal.

### Cell culture

Breast cancer cell line MCF7 and MDA-MB231 were cultured in DMEM supplemented with 10% of foetal calf serum (FCS). Jurkat cells were cultured in RPMI supplemented with 10% of FCS and L-glutamine (2 mM). Peripheral blood mononuclear cells (PBMC) from healthy donors or chronic lymphocytic leukemia (CLL) patients were maintained in RPMI 1640 supplemented with 10% FCS, 1% non-essential amino acids, 1% Hepes, 1% glutamine and 1% sodium piruvate. Cells were maintained at 37°C in humidified 5% CO_2_ atmosphere.

### Isolation of peripheral blood mononuclear cells (PBMC)

Fresh blood samples from healthy donors or chronic lymphocytic leukemia (CLL) patients were obtained from EFS and hematology department, respectively. PBMC were isolated by Ficoll gradient centrifugation. B cells were selected with anti hCD19-APC labelled antibody.

### Structural analysis of peptides

The structure of the peptides was predicted by PEP-FOLD, which is a de novo approach for prediction of peptide structure for peptides in solution.

### Cellular toxicity of the peptides

For viability assay, MDA-MB231, MCF7 or Jurkat cells (5x10^3^/well) were seeded in 96 well plates and treated with the peptide for 4 h or 24 h. After this period, 10 ml of WST-1 cell proliferation reagent (Sigma) was added to each well. Cells were maintained at 37°C for 2h. Absorbance was measured with a FlexStation 3 Multi-Mode Microplate Reader (Molecular Devices) at 450 nm, subtracting absorbance at 640 nm. Measurements were performed in triplicate.

### Detection of apoptosis by Annexin staining

The apoptosis induction of the different shuttles associated to a cargo (PP2A/SET binding site) was analyzed by annexinV-FITC staining (e Biosciences). Human breast cancer cell line MCF7 and human T cell line Jurkat were treated with different concentrations of peptide for 24 h. After the treatment, cells were harvested, washed and treated according to the manufacture-s protocol. The level of apoptosis was measured by flow cytometry (BD Biosciences, FACS Canto). B cells from healthy donor or CLL patients were stained with anti-hCD19-APC labelledantibody (BD Biosciences) after annexin staining.

### Quantification of cellular internalization

Human breast cancer cell line MCF7 and human T cell line Jurkat were seeded in 24 well plates (1x10^5^ cells/well). Cells were treated with different concentrations of FITC-labelled peptides for different periods of time. After the treatment with FITC-labelled shuttles, cells were harvested and washed twice with PBS to remove the extracellular unbound peptide. Cells were treated with trypsin for 5 min to remove non-internalized surface bound peptide and then, centrifuged, washed and resuspended in 200 ml of PBS. FITC fluorescence intensity of internalized peptide was measured by flow cytometry (BD Biosciences) by acquiring 10,000 live cells. Experiments were carried out three times in duplicate. Untreated cells were used as control.

### Peptides internalization visualization

For intracellular localization of FITC-labelled peptides, MDA-MB231 or MCF7 cells were seeded in an 8-well Labtek (Thermo Fischer) at a density of 2x10^4^ cells/well. Cells were treated with different concentrations of FITC-labelled peptides for different times. Cells were fixed or not with 4% of formaldehyde for 15 min at room temperature. Samples were washed twice with PBS and mounted in mounting solution containing DAPI. Images were captured with a fluorescence microscopy (Olympus Japan) using 63x magnification objective.

### Analysis of peptide stability in serum

Peptides were incubated at 37°C in 250 ml of human serum for different periods of time. Samples were collected and peptides degradation stopped by freezing. Peptides were extracted from samples using the Proteo Miner Protein Enrichment System (Bio-Rad). Peptide integrity (percentage of intact peptide) was analyzed by mass spectrometry (MS) using MALDI-TOF (Brucker Autoflex II) following their standard protocols. Measurements were performed in triplicate. MS data were analyzed using appropriate software (Clinprot tools, Flex analysis, Brucker).

### Intracellular detection of peptides by mass spectrometry (MS)

To detect the internalization of the peptides, breast cancer cell line MCF-7 cells were seeded in a 6-well plate at a density of 1 x 10^6^ cells/well in DMEM medium supplemented with 10% FCS. Cells were treated with 100 μM of each shuttle for 4 h at 37 ͦC, then cells were washed once with PBS. Cell pellet was collected by detaching with Trypsin EDTA following several washing step with PBS. Cells were resuspended in lysis buffer (Tris-HCl 50 mM, NaCl 20 mM, pH 8.0, supplemented with protease inhibitors) and lysed with Glass/Teflon potter (Elvehjem homogenizers), then centrifuged for 20 min at 16,000 g at 4 ͦC. The peptides in the supernatant were concentrated with Ziptips (Millpore, ZTC18S096) according to the manufacture’s protocol. The internalization of peptide was detected by mass spectrometry (MS) using a MALDI-TOF (Brucker Autoflx II). MS data were analyzed using appropriate software (Clinprot tools, Flex analysis, Brucker).
